# Significance of bone morphology and quality on the primary stability of orthodontic mini-implants: in vitro comparison between human bone substitute and artificial bone

**DOI:** 10.1007/s00056-022-00385-8

**Published:** 2022-03-18

**Authors:** Sachin Chhatwani, Ouafaa Kouji-Diehl, Kristian Kniha, Ali Modabber, Frank Hölzle, Jozsef Szalma, Gholamreza Danesh, Stephan Christian Möhlhenrich

**Affiliations:** 1 Department of Orthodontics, University of Witten/Herdecke, Alfred-Herrhausen Str. 45, 58455 Witten, Germany; 2 Department of Oral and Maxillofacial Surgery, University Hospital of Aachen, Pauwelsstr. 30, 52074 Aachen, Germany; 3 Department of Oral and Maxillofacial Surgery, University of Pecs, Dischka Győző str. 5, 7621 Pecs, Hungary

**Keywords:** Skeletal anchorage, Resonance frequency analysis, Insertion placement torque, Bone quality, Bone defects, Skelettale Verankerung, Resonanzfrequenzanalyse, Insertionsdrehmoment, Knochenqualität, Knochendefekte

## Abstract

**Aim:**

This study evaluated artificial bone models against a human bone substitute to assess the primary stability of orthodontic mini-implants (OMIs) at varying implant sites with different morphologies and qualities.

**Materials and methods:**

A total of 1200 OMI placements of four types were inserted into four artificial bone models of different density (D1, D2, D3, D4) and into a human bone substitute (HB). The implants varied in diameter (2.0 and 2.3 mm) and length (9 and 11 mm). Each specimen had four implant sites: no defect, one-wall defect, three-wall defect, and circular defect. The implant stability quotient (ISQ) values were measured using resonance frequency analysis (RFA) and insertion placement torque values (IPT) were assessed for primary stability. Correlation analysis was performed to evaluate the different models.

**Results:**

The highest IPT value was registered for the 2.0 mm × 11 mm implant inserted into D1 with no defect (37.53 ± 3.02 Ncm). The lowest ISQ value was measured for the 2.3 mm × 9 mm OMI inserted into D3 with a circular defect (12.33 ± 5.88) and the highest for the 2.3 mm × 9 mm implant inserted into HB with no defect (63.23 ± 2.57). A strong correlation (r = 0.64) for IPT values and a very strong correlation (r = 0.8) for ISQ values was found between D2 and HB.

**Conclusion:**

Bone defects and bone quality affected the primary stability of implants in terms of ISQ and IPT values. Results for bone model D2 correlated very well with the HB substitution material.

**Supplementary Information:**

The online version of this article (10.1007/s00056-022-00385-8) contains supplementary material, which is available to authorized users.

## Abstract (de)

**Ziel:**

In dieser Studie wurden synthetische Knochenmodelle mit einem humanen Knochenersatzmaterial verglichen, um die Primärstabilität kieferorthopädischer Mini-Implantate (OMIs) an verschiedenen Implantatpositionen mit unterschiedlichen Morphologien und Qualitäten zu beurteilen.

**Materialien und Methoden:**

Insgesamt wurden 1200 OMIs von vier verschiedenen Typen in synthetische Knochenmodelle von unterschiedlicher Dichte (D1, D2, D3, D4) und in ein humanes Knochenersatzmaterial (HB) inseriert. Die Implantate variierten im Durchmesser (2,0 und 2,3 mm) und in der Länge (9 und 11 mm). Jedes Exemplar hatte 4 Implantatstellen: kein Defekt, einwandiger Defekt, dreiwandiger Defekt und zirkulärer Defekt. Der Implantatstabilitätsquotient (ISQ), gemessen mit Hilfe der Resonanzfrequenzanalyse (RFA), und die Werte des Insertionsdrehmoments (IPT) wurden für die Primärstabilität bewertet. Zur Evaluation der verschiedenen Modelle wurde eine Korrelationsanalyse durchgeführt.

**Ergebnisse:**

Der höchste IPT-Wert wurde für das 2,0 × 11 mm Implantat registriert, für die Insertion in D1 ohne Defekt (37,53 ± 3,02 Ncm). Der niedrigste ISQ-Wert wurde für das 2,3 × 9 mm OMI gemessen, bei Einbringen in D3 mit einem zirkulärem Defekt (12,33 ± 5,88), und der höchste für das 2,3 × 9 mm Implantat, das in HB ohne Defekt eingesetzt wurde (63,23 ± 2,57). Es wurde eine hohe Korrelation (r = 0,64) für IPT-Werte und eine sehr hohe Korrelation (r = 0,8) für ISQ-Werte zwischen D2 und HB festgestellt.

**Schlussfolgerung:**

Knochendefekte und Knochenqualität beeinflussten die Primärstabilität von Implantaten in Bezug auf ISQ- und IPT-Werte. Die Ergebnisse für das Knochenmodell D2 korrelierten sehr stark mit dem HB-Ersatzmaterial.

## Introduction

Anchorage plays an essential role in orthodontics. During orthodontic treatment, in accordance with Newton’s third law, forces act on the teeth that always result in a reciprocal force of equal magnitude in the opposite direction [23]. Therefore, a desired tooth movement can provoke undesirable side effects and unwanted tooth movements [2]. It is important to avoid these side effects by counteracting opposing forces using an anchorage unit.

The use of orthodontic mini-implants (OMIs) can provide a skeletally anchored anchorage unit.

The failure rates of these OMIs, which usually have a smooth surface, have been variably reported in the literature. According to Pasch, the overall failure rate of OMIs is 10.1%, with interradicularly placed implants having a higher failure rate of 29–40% [37].

The clinical success of skeletal anchorage systems largely depends on primary stability [40], which should be distinguished from secondary stability [20, 43]. Primary stability is derived from a combination of displacement and compaction of bone during implant placement and creates a tight fit between the implant and the bone [24].

Factors such as insertion site preparation, bone quality, insertion angle, and implant design affect primary stability and, thus, the success of implantation [47, 48].

Local patient factors, such as deep bone defects around dental implants, have been associated with higher rates of periodontal pathogenic bacteria [1] and can lead to implant failure due to inflammation [36]. Circular bone defects have also been linked to the loss of primary stability of dental implants [18].

Depending on the anatomic conditions of the underlying bone, the insertion angle, and depth, OMIs cannot always be fully covered with bone, similar to the bone defects described by Goldman and Cohen [16], and this may lead to reduced primary stability.

To our knowledge, the effect of bone defects on OMIs has not been reported. Another crucial factor for stability is bone quality, which can vary within the jaw and depend on gender and age [7].

Primary stability can be reliably measured using resonance frequency analysis (RFA) [31, 41]. The measuring variable resulting from this analysis is the implant stability quotient (ISQ), with higher values indicating higher stability for the implant. Another value that is often assessed with regard to OMI insertion is the insertion placement torque (IPT), which could be higher for thick compact bone [30] but should not exceed 20 Ncm, as higher torque insertion rates could cause implant breakage [47].

In vitro studies evaluating the mechanical properties of OMIs commonly used solid rigid polyurethane foams in different densities to simulate human bone without comparison to human bone specimen [6, 21, 29, 35].

Thus, this in vitro study evaluated artificial bone models against a human bone substitute to assess the primary stability of OMIs at varying implant sites with different bone morphologies and qualities using RFA and IPT measurements. It also examined the effect of peri-implant defect geometry on primary stability.

## Materials and methods

A total of 1200 OMI placements of four different types from the same manufacturer (PSM Medical Solutions, Gunningen, Germany) were inserted into five types of bone models. The implants varied in diameter (2.0 and 2.3 mm) and length (9 and 11 mm). The artificial bone models were made from solid rigid polyurethane foam blocks (#1522-05, #1522-04, #1522-03, #1522‑1, #1522-16; Sawbones, Malmö, Sweden) with different densities: D1 (0.64 g/cm^3^: “very dense bone”), D2 (0.48 g/cm^3^: “dense bone”), D3 (0.32 g/cm^3^: “porous bone”), and D4 (0.16 g/cm^3^: “very porous bone”) [27]. The human bone model (HB) was made up of a block of human femoral bone substitution material (Maxgraft, Botiss Biomaterials, Zossen, Germany), which is considered gold standard. Before inserting the OMIs, the allogeneic bone block was conditioned in sterile saline for 24 h.

For all bone models, regardless of their quality, the same defect preparation according to Shin et al. was carried out [42]. Using trepan drill burs and cylindrical burs, three different defect types were prepared at 4 mm depth and checked with a standard dental probe. Each bone block consisted of four implant sites at a distance of 15 mm: no defect, one-wall defect, three-wall defect, and circular defect.

**Fig. 1:**
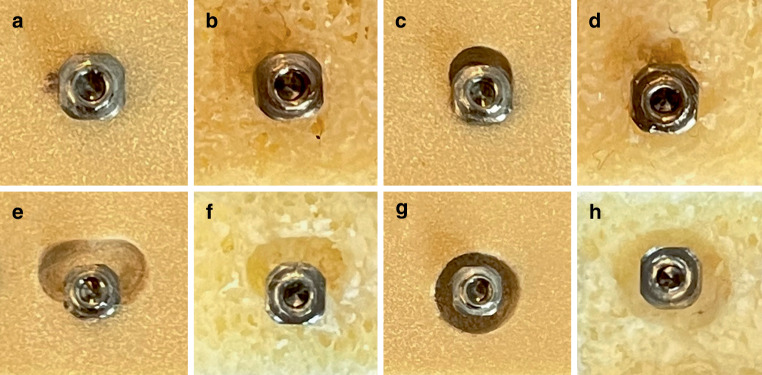
Orthodontic mini-implants (OMI) placed in artificial bone (**a** no defect, **c** three-wall defect, **e** one-wall defect, **g** circular defect) and in human bone allograft (**b** no defect, **d** three-wall defect, **f** one-wall defect, **h** circular defect) Kieferorthopädische Mini-Implantate (OMI) in synthetischem Knochen (**a** kein Defekt, **c** dreiwandiger Defekt, **e** einwandiger Defekt, **g** zirkulärer Defekt) und in menschlichem Knochenallotransplantat (**b** kein Defekt, **d** dreiwandiger Defekt, **f** einwandiger Defekt, **h** zirkulärer Defekt)

In accordance with the various parameters (bone quality, implant length/diameter, and bone defect), 80 groups were obtained. Fifteen implantations were performed in each group. Each implant was used for 15 implantations before being changed but was always inserted into freshly prepared implantation sites (Fig. 1).

For implantation, predrilling was necessary using a surgical unit (Implantmed SI-1023, W&H, Bürmoos, Austria) and twist drills of 1.4 and 1.8 mm in diameter, followed by implantation of the OMIs of 2.0 and 2.3 mm, respectively. The drilling speed was set to 300 rpm, the axial load was 20 N, and the predrilling depth was 5 mm.

Implantation was performed by one investigator, who inserted the OMIs immediately after osteotomy using a surgical handpiece (WS-56L W&H, Bürmoos, Austria) to simulate clinical conditions. The insertion was limited by the length of the implant and was visually controlled by the operator.

Maximum torque was recorded using the surgical unit applied during the insertion of the OMIs.

RFA was conducted using hand-screwed smart pegs (type 8; Osstell, Gothenburg, Sweden) to measure the achieved primary stability. Measurements were carried out in two planes at a 90° angle to each other. For the one- and three-wall defects, the measurement direction was toward the highest bone defect at the implant site, followed by a measurement in the perpendicular direction. The smart pegs were adjusted in consultation with the manufacturer to provide an ideal fit with the internal thread of the OMIs. The ISQ values ranged from 0–100 and were measured between 3500 Hz and 8500 Hz. The values were interpreted as low (ISQ < 60), medium (ISQ = 60–70), and high stability (ISQ > 70), according to the manufacturer [34]. The measurement was repeated three times for each implant. The average values were calculated.

### Statistical analysis

The sample size was calculated using G*Power software (G*Power, Version 3.1.9.2, Düsseldorf, Germany) [13, 14]. The Wilcoxon–Mann–Whitney test for the two groups was used for a priori testing. In an artificial high-density D1 bone, a larger diameter (2.0 mm vs. 2.3 mm) was assumed to significantly increase implant stability. Thus, data from a previous analysis were used [29]. Using a significant level of 0.05, a power of 80%, and an effect size of 2.34 for ISQ (mean ± standard deviation [SD] 1: 55.80 ± 1.36; mean ± SD 2: 59.45 ± 1.74) and 3.15 for IPT (mean ± SD 1: 25.20 ± 0.79; mean ± SD 2: 29.40 ± 1.71), *n* = 4 implants per group for both measuring techniques were needed to verify the hypotheses. To ensure a sufficient effect size for the subgroups, the group size was set to *n* = 10.

After assessment, the values were tested for normal distribution. The factors of the bone model, defect size, implant diameter, and implant length resulted in a 5 × 4 × 2 × 2 factorial design. Multifactorial analysis of variance and post hoc Tukey tests for multiple comparison were used to analyze the groups. A *p*-value less than or equal to 0.05 was considered statistically significant. Correlation analysis according to Spearman’s rho test was conducted to evaluate the ISQ and IPT values. R was considered as very weak (0.00–0.19), weak (0.20–0.39), moderate (0.40–0.59), strong (0.60–0.79), and very strong (0.80–1.0).

## Results

### Insertion placement torque

**Fig. 2:**
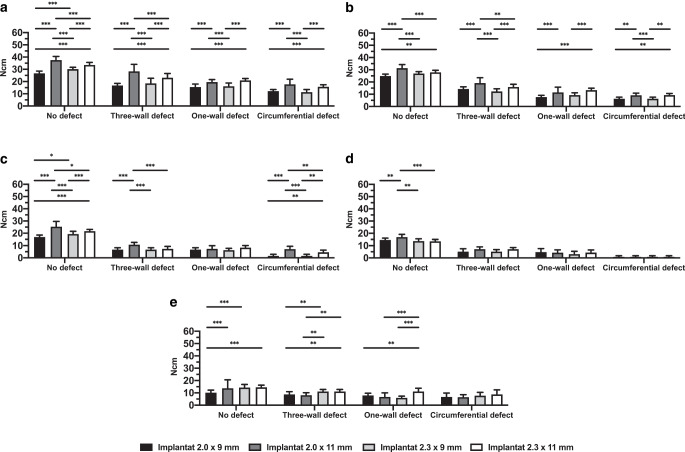
Bar chart with mean values (standard deviation) for insertion placement torque in Ncm depending on bone quality, defect size, implant diameter and implant length. Artificial bone models of different density (**a** D1, **b** D2, **c** D3, **d** D4). Human bone substitute (**e**). ***p < 0.001, **p < 0.005, *p < 0.05 Balkendiagramm mit Mittelwerten (Standardabweichung) für das Eindrehmoment in Ncm in Abhängigkeit von Knochenqualität, Defektgröße, Implantatdurchmesser und Implantatlänge. Synthetische Knochenmodelle mit unterschiedlicher Dichte (**a** D1, **b** D2, **c** D3, **d** D4). Humanes Knochenersatzmaterial (**e**). ***p < 0,001, **p < 0,005, *p < 0,05

The mean values of insertion placement torque (IPT) for the different bone qualities, implant diameters, and implant lengths and for the different types of bone defects are shown in Fig. 2.

The highest IPT value during implantation was registered for the 2.0 mm × 11 mm implant inserted into D1 with no defect (37.53 ± 3.02 Ncm). The lowest insertion torque was found for implantation into D4 with a circular defect and was the same value for all implant types, regardless of length and diameter (0.87 ± 0.92 Ncm).

Primary stability, measured using IPT, was dependent on the defect size. The larger the defect size, the less primary stability could be achieved, except for the circular defect in the HB (7.73 ± 2.66 Ncm). Detailed information on the mean values is presented in Supplementary Table 1.

**Table 1:** Correlations by Pearson test of the insertion torque (Ncm) and implant stability quotient (ISQ) between artificial urethan bone blocks of different qualities (D1–D4) and human bone (HB) substitute Korrelationen (Pearson-Test) des Einsetzdrehmoments (Ncm) und des Implantatstabilitätsquotienten (ISQ) zwischen den künstlichen Urethan-Knochenblöcken unterschiedlicher Qualität (D1–D4) und dem humanen Knochenersatz (HB)

Implant stability		Number of pairs (*N*)	Rank correlation (r)	95% confidence interval	*P* value
Insertion torque (Ncm)	HB vs. D1	240	0.572	0.480–0.651	< 0.001
	HB vs. D2	240	0.636	0.554–0.706	< 0.001
	HB vs. D3	240	0.570	0.478–0.650	< 0.001
	HB vs. D4	240	0.631	0.549–0.702	< 0.001
ISQ	HB vs. D1	240	0.830	0.786–0.866	< 0.001
	HB vs. D2	240	0.806	0.756–0.846	< 0.001
	HB vs. D3	240	0.750	0.689–0.801	< 0.001
	HB vs. D4	240	0.712	0.643–0.769	< 0.001

**Fig. 3:**
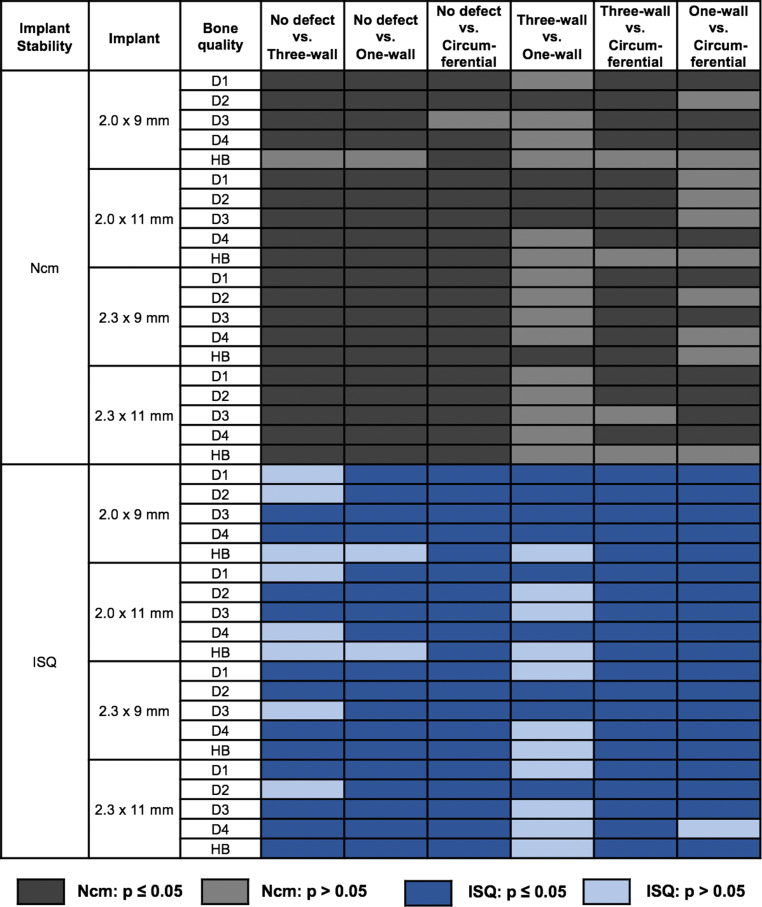
Significance of *p*-values for post hoc Tukey multiple comparison tests for pairwise comparison of bone defect sizes. *D1–D4* Artificial bone models of different density, *HB* human bone, *ISQ* implant stability quotient, *Ncm* for insertion placement torque Signifikanz der *p*-Werte für Post-hoc-Tukey-Mehrfachvergleichstests zum paarweisen Vergleich der Knochendefektgrößen. *D1–D4* Synthetische Knochenmodelle unterschiedlicher Dichte, *HB* humaner Knochen, *ISQ* Implantatstabilitätsquotienten, *Ncm* für das Eindrehmoment

Implants placed in bone defects regardless of implant size compared to the no-defect group generally showed significantly (*p* < 0.05) less primary stability in terms of IPT for all parameters, except for the 2.0 mm × 9 mm implant in the HB. Statistical comparison between the no defect sites and those with a one-wall defect and between the no defect sites and those with a three-wall defect in the HB showed no statistical significance in implant stability for the 2.0 mm × 9 mm implant (*p* > 0.05). A statistically significant difference was found between the no defect sites and those with a circular defect (*p* = 0.02). In the comparison between the one-wall defect sites and those with a three-wall defect, implant stability was higher in the latter one, but this difference was not statistically significant. Comparison between the one-wall defect sites and those with a circular defect showed a heterogeneous pattern for the statistical significances for all parameters (Fig. 3).

**Fig. 4:**
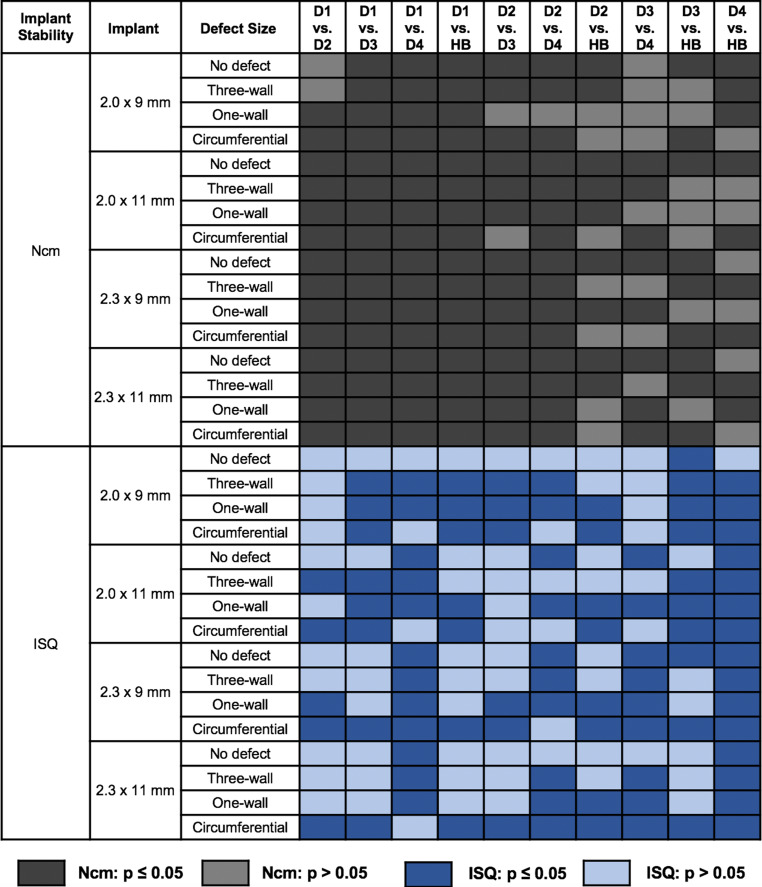
Significance of *p*-values for post hoc Tukey multiple comparison tests for pairwise comparison of bone qualities. *D1–D4* Artificial bone models of different density, *HB* human bone substitute, *ISQ* implant stability quotient, *Ncm* for insertion placement torque Signifikanz der *p*-Werte für Post-hoc-Tukey-Mehrfachvergleichstests zum paarweisen Vergleich der Knochenqualität. *D1–D4* Synthetische Knochenmodelle unterschiedlicher Dichte, *HB* humaner Knochen, *ISQ* Implantatstabilitätsquotienten, *Ncm* für das Eindrehmoment

When comparing D1 with D3/D4/HB, the insertion torque differed significantly for most OMI types and defect sizes (Fig. 4). In general, the measured IPT was significantly higher in D1. Conversely, fewer statistically significant differences were found for the comparisons between D2–D4 and HB.

### ISQ

**Fig. 5:**
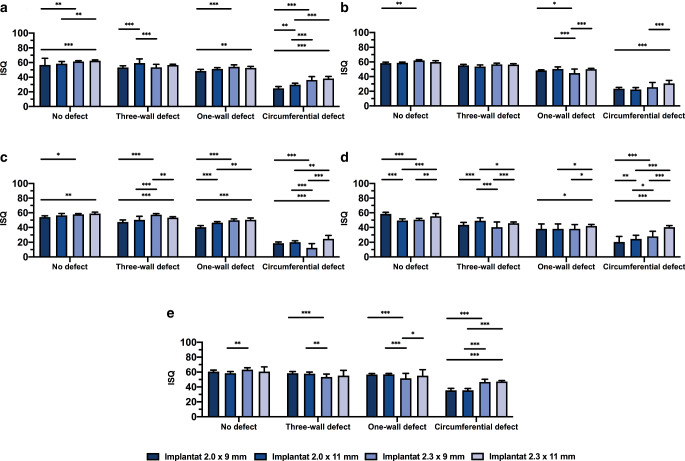
Bar chart with mean values (standard deviation) of implant stability quotient (*ISQ*) depending on bone quality, defect size, implant diameter and implant length. Artificial bone models of different density (**a** D1, **b** D2, **c** D3, **d** D4). Human bone substitute (**e**). ***p < 0.001, **p < 0.005, *p < 0.05 Balkendiagramm mit Mittelwerten (Standardabweichung) des Implantatstabilitätsquotienten (*ISQ*) in Abhängigkeit von Knochenqualität, Defektgröße, Implantatdurchmesser und Implantatlänge. Synthetische Knochenmodelle mit unterschiedlicher Dichte (**a** D1, **b** D2, **c** D3, **d** D4). Humanes Knochenersatzmaterial (**e**). ***p < 0,001, **p < 0,005, *p < 0,05

The primary stability measurement values obtained through RFA are shown in Fig. 5. The results of this measurement showed that the primary stability was lower for the model with circular defects. The lowest ISQ value was measured for the 2.3 mm × 9 mm OMI inserted into D3 with a circular defect (12.33 ± 5.88). The highest value was obtained with a 2.3 mm × 9 mm implant inserted into HB with no defect (63.23 ± 2.57). For artificial bone, the highest value was obtained with a 2.3 mm × 11 mm implant in combination with D1 and no defect.

The combination of D4 and a circular defect was statistically significant (*p* < 0.05) and demonstrated that higher primary stability could be achieved by using an implant with greater diameter, length, or a combination of both.

If relating bone defects to implant stability according to ISQ, the no defect and the three-wall defect implant sites showed significantly higher implant stability for all combinations of bone models and OMIs used in this study when compared with the circular defect sites (*p* < 0.05). The one-wall defect sites showed a higher primary stability (*p* < 0.05) than the ones with a circular defect, except for the 2.3 mm × 11 mm OMI with D4 (*p* > 0.05).

The 2.0 mm × 9 mm OMIs showed higher primary stability in the three-wall defect sites than in the sites with a one-wall defect for D1–D4 (*p* < 0.05) but not for the HB. The comparison of the one-wall defect sites with the ones with a three-wall defect did not show significant differences in the HB (*p* > 0.05) for all implant types.

The *p*-values for ISQ showed lower significant differences for the models with bone quality of higher densities than for the models D1, D2, D3 and HB. Significant differences were found for the ISQ values when comparing D4 with HB for nearly all parameters (*p* < 0.05), except for the 2.9 mm × 11 mm OMI (Fig. 4).

### Correlation of IPT/ISQ

**Fig. 6:**
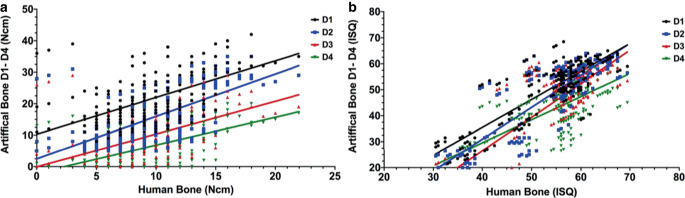
Correlation of insertion placement torque in Ncm (**a**) and implant stability quotient (*ISQ*, **b**) for human bone and artificial bone models Korrelation des Insertionsdrehmoments in Ncm (**a**) und des Implantatstabilitätsquotienten (*ISQ*, **b**) für humanen Knochen und synthetische Knochenmodelle

Spearman’s rank correlation was conducted to evaluate the comparability of HB with the artificial bone models regarding ISQ values and IPT measurements (Table 1). A correlation was found between HB and D2 (r = 0.64) and between HB and D4 (r = 0.63) in terms of IPT. A moderate correlation was found between HB and D1 and D3 (r = 0.57; Fig. 6a). In terms of ISQ, a higher correlation was found between HB and D1 (r = 0.83) and between HB and D2 (r = 0.8), indicating a strong correlation between these bone qualities. A strong correlation was also found between HB and D3 (r = 0.75) and between HB and D4 (r = 0.71) (Fig. 6b; [39]).

## Discussion

The areas for the insertion of OMIs can be the maxillary tuberosity [46], retromolar [38], or palatal [7, 49] region, the mandibular buccal shelf [33], and the interradicular alveolar bone [9].

Exposure of a part of the implant surface is commonly seen in dentistry, especially when inserting dental implants in post-extraction sites [5]. As OMIs can be inserted into the tuberal area after extraction of a wisdom tooth (e.g., for distalization purposes), implant exposure of OMIs also cannot be ruled out, as the tuberal bone is reduced after wisdom tooth extraction [19] or can even fracture during the extraction procedure [8].

The sole application of conventional two-dimensional (2D) radiology for orthodontic implant planning is insufficient for detecting a local defect [15]. A narrow palate, which cannot be assessed using 2D cephalometry, may not allow full utilization of the implant length and therefore may also be a cause of coronal exposure of paramedian-inserted OMIs resembling a circular defect, as it was simulated in our study.

To previsualize implant position and minimize the risk of implant exposure, a 2D cephalogram can be superimposed with a digital three-dimensional model of the patient, and a surgical guide can be created utilizing computer-aided-design/computer-aided-manufacturing procedures [28].

The experimental setup of this study was based on a similar setup used in the study of Ibrahim et al., who investigated dental implants [18]. Primary stability was assessed using IPT and RFA. Both procedures have been applied previously and seem to be appropriate for measuring implant stability [11].

ISQ measurements were taken constantly from the largest defect area of the implant site whenever possible because of differing measurement directions could vary the results [42].

The clinical threshold for ISQ values for the primary stability of dental implants is 62.0 ± 1.1 [4]. A similar value was found in this study for the 2.3 mm × 9 mm implant in the HB with no defect (63.23 ± 2.57). Note that OMIs with values as low as 35.4 ± 2.67 already have shown to present primary stability [31]. These differing values could indicate the necessity of a new ISQ scale adapted to OMIs.

Using RFA measurements, Nienkemper et al. found that 2 mm × 9 mm OMIs provided sufficient primary stability and that there was no statistical difference for an OMI with a length of 11 mm and the same diameter [32]. Other studies also did not show any significant differences in implant stability in soft bone in relation to implant length [45], but the difference of the tested implant lengths was only 1 mm.

In contrast, the current study showed an effect of implant geometry on primary stability. Diameter and length affected the primary stability of the groups with no bone defects for D1, D2, and D3. The IPT measurements showed that the effect of implant length should be higher than that of implant diameter to achieve primary stability. This result was also confirmed by Möhlhenrich et al. [29].

The implant geometry effect seemed to be relevant only in dense bone (*p* < 0.001; D1, circular defect; 2.0 mm × 9 mm vs. 2.3 mm × 11 mm) and seemed to play a minor role in low-density bone (*p* > 0.05; D4, circular defect; 2.0 mm × 9 mm vs. 2.3 mm × 11 mm) if looking at the IPT values. This was confirmed by a decrease in the statistically significant differences for the implants inserted into D1–D4 (Fig. 3) for the IPT. Bayarchimeg et al. also found higher IPT values in bone models of higher density [3]. OMIs with different geometries with a cylindrical or conical design but with the same length and diameter showed no significant difference in performance when inserted into bovine pelvic ilium bone with densities of 0.36 (±0.02) g/cm^3^ and 0.32 (±0.02) g/cm^3^ [10]. This is similar to the D3 model (0.32 g/cm^3^) in our study, which was the only one where significant differences between the implant types were found, indicating that implant length and diameter might affect primary stability. Holm et al. found a significant effect of bone density and OMI diameter on primary stability [17]. With regard to RFA measurements, primary stability was reduced in low-density bone in this experimental setup, consistent with other studies showing a correlation between bone density and primary stability [25].

A study on dental implants revealed that not only defect size but also the location of the defect affected primary stability. A more coronal location of the defect led to reduced primary stability of dental implants [26]. Due to the individual anatomic conditions of the palate or the alveolar ridge and, if necessary, additional oblique angulated insertion, the OMI may not be inserted fully into bone. Therefore, this condition may resemble a coronal defect.

In our study, the ISQ values decreased with an increase of the size of the bone defect. This is consistent with the study of Yao et al., who detected bone defects of 2‑mm depth via a decrease in ISQ values using RFA [50]. Similarly, the IPT measurements decreased for greater bone defects and reduced bone density. These results are in accordance with studies that analyzed dental implants in bone defects [18].

Our findings clearly showed that in the sites with a circular defect in the bone model D4, the implants had significantly lower ISQ values and this result confirmed the effect of implant geometry on the stability of OMIs in sites presenting bony defects and low-density bone. According to the ISQ values, geometry could have a greater effect than the IPT measurement. This fact is evidenced when comparing the pattern of significant values for IPT and ISQ measurements in Fig. 4.

Only isolated significant differences were observed when comparing three-wall and one-wall defects in terms of the pattern of significant values for the IPT. The pattern was similar in terms of the ISQ values when comparing the same defect, but it was clearly different for both measurements if comparing the one-wall and circular defects (Fig. 3). Surprisingly, the IPT values were partially higher for the 2.0 mm OMIs than for the 2.3 mm OMIs. This effect could be due to the different pilot drills affecting stability. A pilot drill with a smaller diameter was shown to be associated with higher success rates in 2.0 mm OMIs [44].

According to our findings, the two methods applied to assess primary stability showed different results. RFA was more sensitive to bone quality with low density, while IPT measurements provided more sensitive information on bone with high density. In this context, Lages et al. reported no correlation between these two measurement methods, which seemed to be independent of one another and not comparable [22].

Bone model D2 demonstrated the highest correlation with the human bone substitute for the IPT measurements, and for model D1 the highest correlation with HB for the RFA measurements was observed. However, the r value for model D2 was only 0.03 smaller, also indicating a very strong correlation with HB.

Therefore, if both methods are regarded, D2 (0.48 g/cm^3^) demonstrated the best correlation with the human bone substitution material. The human mandible owns a typical mean bone density of 1.1 g/cm^3^, and the bone of the anterior maxilla displays a mean density of 0.55 g/cm^3^ in the anterior, 0.31 g/cm^3^ in the posterior, and 0.45 g/cm^3^ in the hard palate [12]. This confirms that the artificial sawbones D2 as well as Maxgraft can be used for OMI experiments to simulate the insertion of OMIs into the hard palate, which is a favorable region for implant insertion [7].

Nevertheless, the results of this study should be evaluated with caution, as in vitro studies have limited transferability to clinical settings.

## Conclusion

Bone defects and bone quality affect the primary stability of implants. It may be favorable to use an implant with greater length and diameter in bone that has lower density. The measurement methods for primary implant stability used in this study have specific sensitivities in different areas. The solid rigid polyurethane foam block D2 is very well comparable in its behavior to the human bone substitute that was used in this study.

### Supplementary Information

**Supplemental Table S1**. Mean values and standard deviation (SD) of implant stability measured by implant stability quotient (ISQ) and insertion torque (IPT) depending on bone quality and defect size

## Data Availability

All data are available upon request.
