# Supplementary material for: Significance of bone morphology and quality on the primary stability of orthodontic mini-implants: in vitro comparison between human bone substitute and artificial bone
Source: J Orofac Orthop. 2022 Mar 18;84(6):362–72. doi: 10.1007/s00056-022-00385-8 (PMC10587204; doi:10.1007/s00056-022-00385-8)
Supplement: Supplementary file 1 — Supplemental Table S1. Mean values and standard deviation (SD) of implant stability measured by implant stability quotient (ISQ) and insertion torque (IPT) depending on bone quality and defect size [file 56_2022_385_MOESM1_ESM.pdf]

Supplemental Table 1. Mean values and standard deviation (SD) of implant stability measured by implant stability quotient (ISQ) and insertion torque (IPT) depending on bone quality and defect size

**Bone quality**	**Defect size**	**Implant 2.0 x 9 mm**				**Implant 2.0 x 11 mm**				**Implant 2.3 x 9 mm**				**Implant 2.3 x 11 mm**			
		**IPT (Ncm)**		**ISQ**		**IPT (Ncm)**		**ISQ**		**IPT (Ncm)**		**ISQ**		**IPT (Ncm)**		**ISQ**	
		Mean	SD	Mean	SD	Mean	SD	Mean	SD	Mean	SD	Mean	SD	Mean	SD	Mean	SD
**D1**	**No defect**	26.73	1.79	56.73	9.06	37.53	3.02	58.43	2.98	30.13	1.51	61.43	1.07	33.53	2.10	62.43	1.21
	**Three-wall**	16.73	1.79	53.23	2.31	28.33	5.74	59.33	5.62	18.53	4.16	53.53	4.01	23.13	3.42	56.63	1.08
	**One-wall**	15.53	2.39	48.43	2.21	19.53	2.10	51.33	1.64	16.13	2.64	54.13	2.75	20.93	1.53	52.63	2.17
	**Circumferential**	12.13	1.36	24.83	2.45	17.73	4.22	29.73	1.98	11.33	2.16	36.13	4.73	15.73	1.67	38.13	2.94
**D2**	**No defect**	24.93	1.53	58.33	1.44	31.33	2.92	58.83	1.05	26.93	1.53	62.13	1.03	27.93	1.67	59.83	1.91
	**Three-wall**	14.33	1.72	55.33	1.40	19.13	4.41	53.73	2.19	12.33	2.16	56.73	1.62	15.93	2.22	56.33	1.28
	**One-wall**	7.73	1.39	48.33	0.88	11.53	4.26	50.43	2.81	9.33	1.84	44.73	5.56	13.33	1.59	50.13	1.17
	**Circumferential**	6.33	1.29	23.53	1.76	9.13	1.77	22.33	2.68	6.33	1.29	25.53	6.52	9.33	1.29	30.73	4.06
**D3**	**No defect**	16.93	1.53	54.03	1.76	25.33	4.34	56.53	2.53	19.33	2.26	57.93	1.02	21.73	1.39	58.93	2.01
	**Three-wall**	6.73	1.53	47.33	2.82	10.73	1.91	50.33	4.89	6.73	1.53	57.43	1.52	7.33	2.06	53.33	1.28
	**One-wall**	6.73	1.53	40.23	2.16	7.33	2.61	46.53	1.38	6.33	1.45	49.73	1.98	8.33	1.72	50.43	2.42
	**Circumferential**	1.53	1.36	18.53	1.76	7.13	2.29	20.13	1.88	1.53	1.36	12.33	5.88	4.53	1.77	24.73	4.65
**D4**	**No defect**	14.53	1.51	58.53	2.12	16.93	2.22	49.53	2.19	13.73	1.79	50.53	1.76	13.53	1.51	55.23	3.70
	**Three-wall**	5.13	2.39	43.53	3.29	7.13	1.88	49.23	3.87	5.13	1.64	40.33	7.17	7.13	1.36	45.73	1.75
	**One-wall**	4.80	2.83	38.13	6.65	4.27	2.31	38.13	6.65	3.07	2.31	38.13	5.86	4.33	2.16	41.93	2.14
	**Circumferential**	0.87	0.92	20.33	7.57	0.87	0.92	24.53	4.99	0.87	0.92	28.03	6.78	0.87	0.92	40.33	2.25
**HB**	**No defect**	10.13	2.10	60.53	2.17	13.73	6.85	58.53	2.12	14.33	2.53	63.23	2.57	14.53	1.77	60.73	6.22
	**Three-wall**	8.73	2.22	58.53	2.12	8.13	2.00	57.73	2.28	11.13	1.64	53.33	3.96	11.13	1.64	55.23	7.13
	**One-wall**	7.93	1.79	56.73	1.33	6.73	3.24	56.83	1.32	5.93	1.39	51.63	6.66	11.13	2.64	55.13	8.05
	**Circumferential**	6.73	3.10	35.43	2.69	6.53	2.00	35.53	2.38	7.73	2.66	46.73	3.59	8.73	3.73	47.33	1.28
